# The effect of *Helicobacter pylori* treatment on remission of idiopathic central serous chorioretinopathy

**Published:** 2011-01-11

**Authors:** Mohammad Bagher Rahbani-Nobar, Alireza Javadzadeh, Leila Ghojazadeh, Mandana Rafeey, Amir Ghorbanihaghjo

**Affiliations:** 1 Department of Ophthalmology, Tabriz University of Medical Sciences, Tabriz, Iran; 2 Liver and gastrointestinal research center, Tabriz University of Medical Sciences, Tabriz, Iran; 3 Biotechnology research center, Tabriz University of Medical Sciences, Tabriz, Iran

## Abstract

**Purpose:**

The aim of this study was to evaluate the effect of *Helicobacter pylori* (*H*. *pylori*) treatment on remission of idiopathic central serous chorioretinopathy.

**Methods:**

Twenty-five patients with idiopathic central serous chorioretinopathy (ICSCR) who were infected with *H*. *pylori* were treated with an anti-*H*. *pylori* treatment; another twenty-five patients with the same clinical presentations served as the control. Baseline examination and follow up visits at 2, 4, 6, 8, 12, and 16 weeks after the onset of treatment included visual acuity testing and subretinal fluid measurement. The difference between mean visual acuity at the end of 16 weeks and the time of subretinal fluid reabsorption was compared between the two groups.

**Results:**

Subretinal fluid reabsorption time was 9.28±3.20 weeks in the treatment group and 11.63±3.18 weeks in the control group, which was statistically significant (p=0.015). After 16 weeks, mean visual acuity improved to 0.003±0.01 (logMAR) in the treatment group and 0.004±0.02 (logMAR) in the control group. This improvement did not represent a statistically significant difference (p=0.97).

**Conclusions:**

An anti-*H*. *pylori* treatment regimen is effective in the treatment of idiopathic central serous chorioretinopathy patients and anti-*H*. *pylori* treatment can provoke the faster reabsorption of subretinal fluid.

## Introduction

Typically, idiopathic central serous chorioretinopathy (ICSCR) is defined as a neurosensory serous retinal detachment of unknown origin that affects the macula. It preferentially afflicts young and middle-aged adults and occurs in men more often than in women [1]. Recurrences have been documented in 30%–50% of cases [2,3]. The precise pathophysiology of ICSCR is still poorly understood, although sympathomimetic agents [4], systemic corticosteroid therapy [5], type A characters [6], uncontrolled systemic hypertension, pregnancy, and allergic respiratory disease [7] have been reported as potential associated risk factors for the disease. The disease often resolves spontaneously, but sometimes recurs or becomes chronic [8,9]. Piccolino et al. [10] demonstrated changes in the foveal photoreceptor layer in ICSCR that were highly correlated with loss of visual acuity after macular reattachment. The photoreceptors are expected to die when detachment separates them from the retinal pigment epithelium and the choriocapillaries, their source of oxygen and nutrients. A long duration of ICSCR is associated with macular zone photoreceptor loss and limited visual prognosis after reattachment. There is not a definitive treatment for ICSCR and counseling alone does not satisfy most young patients; thus, some form of medication, such as tranquilizers and β-blockers, are administered and some patients are treated by laser photocoagulation, as that is the only effective palliative measure. Laser photocoagulation may be considered for cases in which ICSCR persists beyond 3–4 months. However, laser therapy has certain limitations, for example, the leakage site is too close to the center of the fovea for laser photocoagulation in some patients and some patients have post laser choroidal neovascularization (CNV) complications. The method of properly treating ICSCR to shorten the course of serous detachment remains to be determined because the precise etiology of disease is unknown. *Helicobacter pylori* (*H*. *pylori*) is a Gram-negative bacterium of spiral appearance that is associated with multiple digestive and extra digestive pathologies [11]. *H*. *pylori* gastric infection has been implicated as an important factor in occlusive arterial pathology. It is suspected that ICSCR is due to a multifocal vascular occlusive disease of choriocapillaries. Currently, the possible physiologic relationship between ICSCR and *H*. *pylori* is a controversial topic on which several studies have been published in recent years [12]. A case report of ICSCR in a 43 year-old man documented that recurrences of the disease were always associated with the presence of *H*. *pylori* and resolution of ICSCR and recovery of visual acuity were correlated significantly with successful eradication of the bacterium using conventional antimicrobial triple therapy (amoxicillin, clarithromycin, and omeprazole) [13]. This paper aims at establishing a possible pathogenic relationship between gastric *H*. *pylori* infection and ICSCR. The current study evaluates the effect of *H*. *pylori* treatment on remission of ICSCR.

## Methods

During a randomized case controlled clinical trial study, we evaluated a group of patients with ICSCR who were referred to our referral center, the Nikookari Eye Hospital—the department of ophthalmology at Tabriz University of Medical Sciences—over a period of 23 months between February 2008 and January 2010.

Clinical ICSCR diagnosis was confirmed by optical coherence tomography (OCT) and fluorescein angiography. The diagnosis was established based on neurosensory detachment with one or more sites of leakage at the level of the retinal pigment epithelium (RPE) without other causes of exudation, such as CNV, inflammation, or infiltration.

We evaluated a total of 58 patients with ICSCR. Among these patients, 50 patients who had ICSCR, were infected with *H*. *pylori,* and met our inclusion criteria were included in the study. The patients were divided in two groups using random allocation software. *H*. *pylori* infection was assessed by the C13-urea breath test. At the end of treatment course, patients who received *H*. *pylori* eradication regiment underwent urease breath test (UBT) testing to evaluate the effectiveness of the anti-*H*. *pylori* treatment. The inclusion criteria were as follows: To accept participation in the study by signing an informed consent; not having been treated with antibiotics, a proton pomp inhibitor, corticosteroids, or sympathomimetic drugs for 3 months before the study; and having no history of previous ocular surgery. Twenty-five patients were treated with an *H*. *pylori* eradication regiment including metronidazole and amoxicillin 500 mg three times daily for two weeks and omeprazole once daily for 6 weeks. Another 25 patients served as a control group and received no medication. The patients who had persistent subretinal fluid after 16 weeks received laser photocoagulation therapy. Baseline examination and follow-up visits at 2, 4, 6, 8, 12, and 16 weeks after the onset of treatment included testing of best corrected visual acuity and evaluation of the amount of the neurosensory or RPE detachment, measured by OCT (Stratus OCT3; Carl Zeiss Meditec Inc., Dublin, CA). The latter was determined by manually placing the calipers and using the scan with the highest serous retinal detachment area.

### Statistical analysis

Independent samples *t*-test, χ^2^, or Fishers Exact test were used to compare results between the groups, and the Weibull survival model was used to determine the early response to treatment. All statistical tests were two-sided. A P value of 0.05 was considered statistically significant. Statistical analysis was performed using the statistical package SPSS.15/win. This study has been cited at the Clinical trial website (ID: NCT00817245).

## Results

Mean patient age was 32.54±4.57 years old (range 24–41) in the treatment group and 34.24±4.78 years old (range 27–43) in the control group (p=0.20). The female to male ratio was 4/21 in the treatment group and 5/20 in the control group. Mean interval between beginning of symptoms and treatment was11.96±8.15 days in the treatment group and 9.20±6.50 days in the control group (p=0.18). All patients responded to eradication treatment and the results of the urea breath test were negative in all patients in the treatment group. No systemic adverse effects of medication were observed.

In the treatment group, 23 patients had pure neurosensory detachment and 2 patients showed both neurosensory and RPE detachment. In the control group, 22 patients had pure neurosensory detachment and 3 patients showed both neurosensory and RPE detachment.

**Figure 1:**
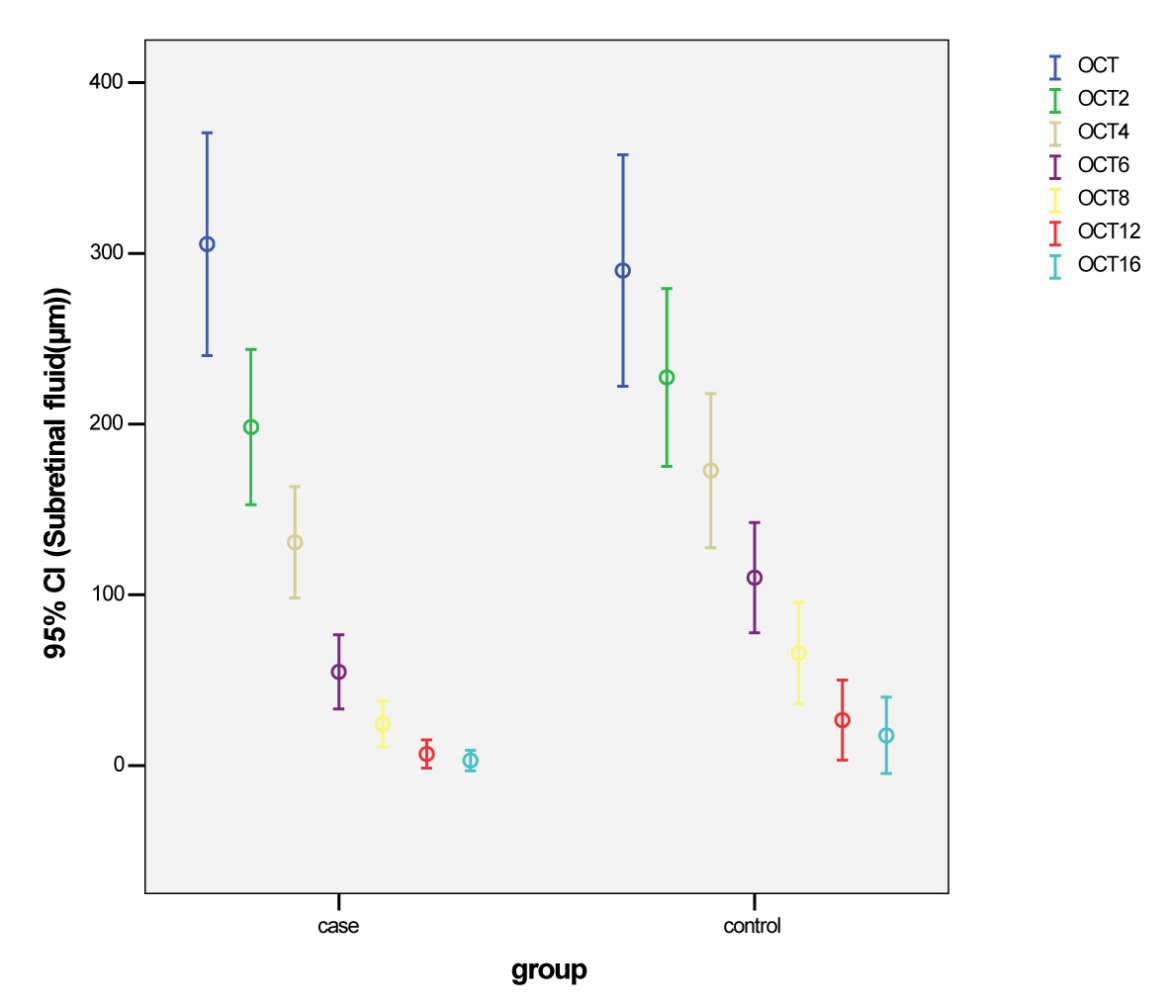
The trend of subretinal fluid reduction measured by optical coherence tomography at baseline and follow up visits at 2, 4, 6, 8, 12, and 16 weeks after the onset of treatment. The sample size was 25 cases for treatment group and 25 cases for control group throughout the study.

Average neuroretinal and/or pigment epithelial detachment at baseline was 305.45±161.48 µm in the treatment group and 290.00±164.38 µm in the control group. The differences at baseline were not statistically significant (p=0.73); the subretinal fluid reduction trend is presented in Figure 1 and Table 1. The subretinal fluid reabsorption time was 9.28±3.20 weeks in the treatment group and 11.63±3.18 weeks in the control group, which was statistically significant (p=0.015). The Weibull survival model showed that the time of response to medication was statistically different between the two groups (p=0.04); in the treatment group, subretinal fluid reached zero earlier than the control group. Laser therapy was performed due to persistent subretinal fluid in 3 control group patients and 1 treatment group patient (Table 2).

**Table 1:** Mean subretinal fluid measured by optical coherence tomography at baseline and follow up visits at baseline 2, 4, 6, 8, 12, and 16 weeks after the onset of treatment.

**Follow up (weeks)**	**Subretinal fluid level (µm)**		**p-value**
**Case (n=25)**	**Control (n=25)**
**0**	305.46±161.48	290.00±164.38	0.73
2	198.31±112.51	227.32±126.12	0.39
4	130.77±80.50	172.76±109.28	0.12
6	54.88±53.95	110.04±78.28	0.21
8	24.35±33.70	65.84±72.05	0.11
12	6.81±20.41	26.64±6.70	0.1
16	2.92±14.90	17.68±54.36	0.18

*Data are presented as Mean±SD.

**Table 2:** Number of cases that reach to zero sub retinal fluid value in cases and controls groups*

**Follow up visit (weeks)**	**Case**	**Control**	**p-value**
2	0 (0)	0 (0)	-
4	0 (0)	0 (0)	-
6	7 (28%)	2 (8%)	0.07
8	14 (56%)	5 (20%)	0.02
12	22 (88%)	17 (68%)	0.09
16	24 (96%)	22	0.35

*Data are presented as number (percentage). *Only at 8th week of follow up the difference between two groups was statistically significant.

At baseline, mean visual acuity (VA) was 0.10±0.05 (logMAR) in the treatment group and 0.09±0.05 (logMAR) in the control group. The differences were not clinically significant (p=0.83). Sixteen weeks later, mean VA improved to 0.003±0.01 (logMAR) in the treatment group and 0.004±0.02 (logMAR) in the control group. This improvement did not represent a statistically significant difference (p=0.97).

## Discussion

Idiopathic central serous chorioretinopathy is a macular serous detachment secondary to one or more focal lesions of the RPE, typically affects young men, and usually resolves with good visual prognosis, but sometimes can become chronic and progressive with severe visual loss [14].

The pathogenesis and treatment of ICSCR is still poorly understood. The disease is characterized by breakdown of the outer retinal barrier, with leakage of fluid through a defect in the retinal pigment epithelium into the subretinal space, resulting in a serous neurosensory detachment [15].

A correlation between ICSCR and *H*. *pylori* infection has recently been hypothesized [12-16]. This association is still unclear. A possible explanation might arise indirectly from the published hypothesis of a pathogenic link between *H*. *pylori* infection and atherosclerosis. A cross-reactivity of anti-cagA antibodies with vascular wall antigen has been postulated in another study, and the immunoglobulin G antibody response to multiple pathogens has been considered as an independent risk factor for endothelial dysfunction [17-19]. The association between coronary artery disease and several infectious pathogens such as *H*. *pylori* was found to be modulated by the interleukin (IL)-6/G-174C polymorphism, this interaction being mediated by variations in serum IL-6 levels [20].On the other hand, a decreased foveal choroidal blood flow has been demonstrated by laser Doppler flowmetry and using fluorescein and ICG angiography with confocal scanning laser ophthalmoscopy. These non-perfuse areas probably result from filling delays of the choroidal arteries and the choriocapillaries [21]. The already mentioned focal occlusion of the choroidal microcirculation together with interaction between *H*. *pylori* and vascular endothelium could explain the mechanism of choroidal ischemia and the development of ICSCR in *H*. *pylori* infected patients. In a prospective study, with 16 ICSCR patients in the south of France, Mauget-Faysse et al. [22] detected *H*. *pylori* infection in 56.3% of patients. This percentage is much higher than the 27.5% of the control group.

The other complementary study, which was done by Ahnoux-Zabsone et al. [23], aimed to verify the results of Mauget-Faysse’s study. The difference in the prevalence of *H*. *pylori* between the study population (39.7%) and the overall population of 58,419,710 inhabitants of France in 1999 (25.4%) was found statistically significant.

Asensio-Sachez et al. [24] showed a possible statistical association between *H*. *pylori* gastric infection and ICSCR. In their study, the difference in the prevalence of *H*. *pylori* between the ICSCR group (68.75%) and the control group (30%) was found to be statistically significant (p<0.05), and they regarded *H*. *pylori* infection as a risk factor in ICSCR patients.

In cases of recent onset, central vision is minimally affected and usually returns to normal after reabsorption of the subretinal fluid. In recurrent and chronic cases, progressive and irreversible visual decline can be associated with the development of central retinal pigment epithelium atrophy, cystoid macular degeneration, and foveal atrophy [8,9]. We selected the acute cases of ICSCR with no history of chronic or recurrent ICSCR. There was no cystoid change at the macula due to chronicity, and this may result in no difference in visual acuity between the two groups; however, the subretinal fluid reabsorption time was greater in the control group than the treatment group, which was statistically significant and will play a significant role in the outcome of disease since a long duration of ICSCR is associated with macular zone photoreceptor loss [10]. On the other hand, in 3 control group patients and 1 treatment group patient, laser therapy was performed due to persistent subretinal fluid.

All of the above-mentioned studies showed that *H*. *pylori* could be a risk factor in patients with ICSCR. In this study, our results showed that an anti-*H*. *pylori* treatment regimen is effective in the treatment of ICSCR patients and that anti-*H*. *pylori* treatment can provoke the faster reabsorption of subretinal fluid. Thus, upon these results, the association between *H*. *pylori* and ICSCR became stronger than previously thought. This new finding could lead to a new therapeutic approach to ICSCR.

We recommend further studies with larger numbers of patients, longer follow-up periods, and use of other functional tests, such as color vision, Amsler, Watzke-Allen, to evaluate the efficacy of *H*. *pylori* treatment on ICSCR.
